# Heterozygosity for Fibrinogen Results in Efficient Resolution of Kidney Ischemia Reperfusion Injury

**DOI:** 10.1371/journal.pone.0045628

**Published:** 2012-09-19

**Authors:** Amrendra Kumar Ajay, Janani Saikumar, Vanesa Bijol, Vishal S. Vaidya

**Affiliations:** 1 Renal Division, Department of Medicine, Brigham and Women’s Hospital, Harvard Medical School, Boston, Massachusetts, United States of America; 2 Department of Pathology, Brigham and Women’s Hospital, Harvard Medical School, Boston, Massachusetts, United States of America; 3 Department of Environmental Health, Harvard School of Public Health, Boston, Massachusetts, United States of America; Goethe University, Germany

## Abstract

Fibrinogen (Fg) has been recognized to play a central role in coagulation, inflammation and tissue regeneration. Several studies have used *Fg* deficient mice (*Fg^−/−^*) in comparison with heterozygous mice (*Fg^+/−^*) to point the proinflammatory role of Fg in diverse pathological conditions and disease states. Although Fg^+/−^ mice are considered ‘normal’, plasma Fg is reduced to ∼75% of the normal circulating levels present in wild type mice (*Fg^+/+^*). We report that this reduction in Fg protein production in the *Fg^+/−^* mice is enough to protect them from kidney ischemia reperfusion injury (IRI) as assessed by tubular injury, kidney dysfunction, necrosis, apoptosis and inflammatory immune cell infiltration. Mechanistically, we observed binding of Fg to ICAM-1 in kidney tissues of *Fg^+/+^* mice at 24 h following IRI as compared to a complete absence of binding observed in the *Fg^+/−^* and *Fg^−/−^* mice. Raf-1 and ERK were highly activated as evident by significantly higher phosphorylation in the *Fg^+/+^* kidneys at 24 h following IRI as compared to *Fg^+/−^* and *Fg^−/−^* mice kidneys. On the other hand Cyclin D1 and pRb, indicating higher cell proliferation, were significantly increased in the *Fg^+/−^* and *Fg^−/−^* as compared to *Fg^+/+^* kidneys. These data suggest that *Fg* heterozygosity allows maintenance of a critical balance of Fg that enables regression of initial injury and promotes faster resolution of kidney damage.

## Introduction

Fibrinogen (Fg) is a 340 kDa glycoprotein, a homodimer linked by disulphide bonds with each unit comprising of 3 distinct polypeptide chains (Aα, Bβ and γ) that are encoded by 3 separate genes (*FGA*, *FGB* and *FGG*) [1]. Apart from its prominent role in the coagulation cascade, Fg serves as an acute phase response protein by acting as a ligand for receptors expressed on cells recruited to the site of inflammation [2]. In humans, several polymorphisms have been described most of them clustered in the *FgB* gene [3] resulting in chronically elevated levels of Fg [4], [5]. Hyperfibrinogenemia (characterized by high circulating plasma levels of Fg) is consistently associated with an increased risk of cardiovascular diseases [6]; conversely, afibrinogenemia causes severe hemorrhagic risks in affected patients [7]. This suggests the necessity to maintain a critical balance in the levels of Fg that is high enough to maintain adequate clot formation yet low enough to reduce its interactions with cellular receptors along with reducing the availability of fibrin matrices that act as centers of migration and proliferation of immune and endothelial cells in instances of acute and chronic inflammation.

The contribution of Fg in disease pathophysiology of various organs has been studied using Fg deficient mice (Fg^−/−^) that lack the Aα chain, which precludes assembly of functional circulating protein [8], [9]. Fg deficient mice (*Fg^−/−^*) that lack the Aα chain [8] have shown to be protected from variety of injury/disease states such as atherosclerosis [10], colitis [11], crescentric glomerulonephritis [12], Duchenne muscular dystrophy [13], endotoxemia [14], fibrosis [15], [16], multiple sclerosis [17], myocardial ischemia-reperfusion injury [18], ischemic neurodegeneration [19] and rheumatoid arthritis [20]. It should be noted that all of these studies used littermate *Fg^+/−^* mice as experimental controls and not as experimental groups for comparison. Although the *Fg^+/−^* mice do not show any symptoms of abnormal clotting and are for all purposes ‘normal’ when compared to *Fg^−/−^* mice, the plasma level of the Fg protein is reduced to ∼75% of the normal circulating levels present in wild type mice (*Fg^+/+^*) [9]. The reduction in Fg may not be significant enough to impair the coagulation cascade but could still suffice to alter the binding response to various cellular receptors thereby transforming the immune system’s inflammatory response.

The objective of our study, therefore, was to evaluate the expression profile of Fg following kidney ischemia reperfusion injury (IRI) and to characterize the phenotype of the *Fg^−/−^* and *Fg^+/−^* mice against animals homozygous for the Aα gene (*Fg^+/+^*) in the context of kidney IRI.

## Methods

### Ethics

All animal maintenance and treatment protocols were in compliance with the Guide for Care and Use of Laboratory animals as adopted and promulgated by the National Institutes of Health and were approved by the Harvard Medical School Animal Care and Use Committees (IACUC).

### Animals

Littermate male wild type (*Fg^+/+^*), heterozygous *(Fg^+/−^*) and knock out (*Fg^−/−^*) mice for fibrinogen on *BALB/c* background (25–29 g) were used for the experiment [9]. Dr. Jay L. Degen at Children’s Hospital Research Foundation, Cincinnati, Ohio, kindly provided breeding pairs of genetically modified Fg mice. Neonate mice experience spontaneous bleeding events, which proves fatal only in 30–50% of cases (depending on strain) and those who survive display otherwise normal organ physiology [9].

### Experimental Design

In the first set of experiments twenty male *BALB/c* mice were anesthetized using pentobarbital sodium (30 mg/kg, ip) and subjected to 25 min bilateral IRI [21] for characterization of fibrinogen expression and excretion. Mice were sacrificed at 24, 48 and 72 hours after reperfusion (n = 5/timepoint). In the next set of experiments genetically manipulated mice (54 male wild type, heterozygous and knockout mice) were anesthetized as mentioned above and subjected to 29 min of bilateral renal I/R surgery by the retroperitoneal approach. Sham surgery was performed with exposure of both kidneys but without induction of ischemia. Mice (n = 6/group/timepoint) in the respective groups (sham or I/R) were injected with BrdU (50 mg/kg, ip) 3 hr prior to sacrifice. Mice were sacrificed at 12 and 24 h following reperfusion using overdose of pentobarbital (180 mg/kg, ip).

Serum creatinine (SCr) concentrations and blood urea nitrogen (BUN) were measured using a VetScan VS2 (Abaxis, Union City, CA). Plasma Fg (D-Dimer) test was performed by Asserachrom D-Di ELISA kit from Diagnostica Stago, Inc. (Parsippany, NJ) as per manufacturer’s instruction. Urinary Fg levels were measured using commercially available Luminex assay based kit from Millipore (Billerica, MA). Urinary creatinine concentration was used to normalize fibrinogen in order to account for the influence of urinary dilution.

### Real Time PCR

Total RNA was extracted by TRIzol reagent (Invitrogen Corporation) as per manufacturer’s protocol. Forward and reverse primer sequences for mouse specific genes were designed using MacVector software (MacVector Inc., Cary, NC) and are listed in Table S1.

### *In situ* Hybridization

Kidney, liver and heart cryosections were washed with TBS and *in situ* hybridization was performed with universal digoxigenin based *in situ* hybridization and detection kit as per manufacturer’s instructions (Invitrogen, Carlsbad, CA). The probe sequences used are as follows: Fgα-AAT ATG CAA AGA TAG GCA TCA CCC AGA TTG AAG TAG CTA CTG CCT ACC TGC CTG T; Fgβ-AGT ATA CTC TGT ACG GCT TGA TGG AGG TGT CAG GCT GGA TGA GAT ACA TTT CGG A; Fgγ-CTC AGT GCA TAT GGA ATT GTG GAC TGC ATG CTT ATC AAA TGA ATC TTC TCA TTT C.

### Immunofluorescence Staining

Immunostainings in the frozen kidney sections was performed using rat monoclonal anti-F4/80 a kind gift from Dr. Bonventre’s laboratory, BWH, Boston, MA and rat monoclonal anti-BrdU (Cell Signaling Technology, Danvers, MA). The primary antibody was detected using donkey anti rat Cy3 labeled and donkey anti rat FITC labeled secondary antibodies respectively (Jackson ImmunoResearch Laboratories, West Grove, PA). DAPI (Sigma Aldrich, St. Louis, MO) was used for nuclear staining. Apoptosis was measured in kidney tissues by TUNEL assay using the *In Situ* Cell Death detection kit (Roche Applied Science) according to manufacturer’s instructions [22]. The images were captured by Nikon DS-Qi1Mc camera attached to Nikon eclipse 90i fluorescence microscope using oil immersion objective 60/1.4 NA by Nikon NIS elements AR ver 3.2 software.

### Immunoblotting and Immunoprecipitation

Kidney tissues were homogenized in RIPA buffer [50 mM Tris-HCl pH 7.4, 150 mM NaCl, 1% NP40, 1 mM PMSF, 1 mM NaF, 20 mM Na_4_P_2_O_7_, 2 mM Na_3_VO_4_, 1X protease inhibitor cocktail (Roche Applied Science, Indianapolis, IN)] and equal protein (30 µg) was resolved by polyacrylamide gel electrophoresis. For plasma 0.02 µl was loaded on the gel. Proteins were transferred onto nitrocellulose membrane and western blotting was performed with rabbit polyclonal anti-fibrinogen (Dako), mouse monoclonal anti-pERK, anti-ERK2 (BD Biosciences San Diego, CA), anti-Cyclin D1, anti-pRb, anti-β -Actin (Cell Signaling Technology), anti-α-Tubulin (Sigma) and goat polyclonal HRP conjugated anti-mouse albumin (Abcam, Cambridge, MA). HRP conjugated secondary antibodies against mouse, rabbit and goat was purchased from Jackson Immunoresearch (West Grove, PA). For Immunoprecipitation (IP) tissues were lysed in IP buffer (20 mM Tris-HCl pH 8.0, 137 mM NaCl, 10% glycerol, 1% NP-40, 2 mM EDTA) containing protease inhibitor cocktail and 300 µg protein was incubated overnight at 4°C with 4 µg of rabbit polyclonal anti-fibrinogen antibody (Dako). Fifty microlitre of protein A/G agarose was added and incubated for 2 h at room temperature. Beads were washed thrice with IP buffer. Immune complex was eluted by adding 1X SDS loading dye and heating at 100°C and western blot was performed to detect ICAM-1 (goat polyclonal, R&D Systems) and goat polyclonal anti-fibrinogen (Nordic lab, The Netherlands).

### Statistics

Data are expressed as average + standard error. Statistical difference (p<0.05) as calculated by one-way ANOVA or student’s t-test. P<0.05 was considered significant and represented by ‘*’ as compared to shams, ‘^#^’ as compared to wild type at similar time points, ‘^!^’ as compared to heterozygous at similar time points where applicable. All graphs were generated by GraphPad Prism (GraphPad, Inc., La Jolla, CA).

## Results

**Figure 1:**
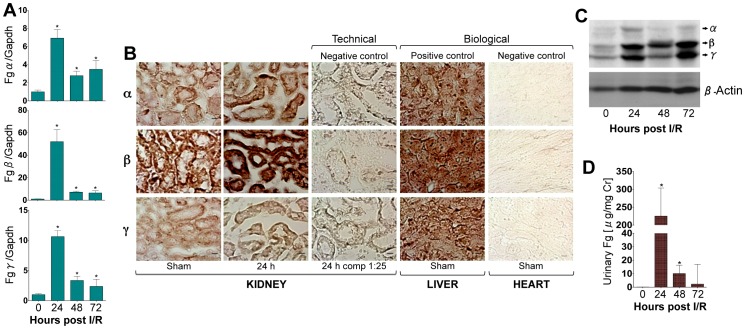
Transcription and translation of *Fgα*, *Fgβ* and *Fgγ* in the kidney as urinary Fg excretion is significantly increased following IRI in mice. To characterize the *de novo* expression of Fg at the mRNA and protein level in the kidney, male *BALB/c* were subjected to IRI and kidneys, blood and urines were collected over time (n = 5/time point). A) Real time PCR analysis in kidney for *Fgα*, *Fgβ* and *Fgγ* chains, normalized to GAPDH, and fold change determined over sham. B) *In situ* hybridization for *Fgα*, *Fgβ* and *Fgγ* mRNA in kidney, 1∶25 represents competition with unlabeled probe, liver (positive control) and heart (negative control). C) Western blot analysis for Fg protein in the kidney. β-Actin served as loading control. D) Urinary Fg levels measured by Luminex assay. *represents p<0.05 as determined by student’s t-test as compared to sham. Bar represents 10 µm.

We found a significant increase in the mRNA (Fig. 1A, 1B), protein expression (Fig. 1C) of Fg (*Fgα*, *Fgβ* and *Fgγ*) in the kidney and urinary excretion of Fg (Fig. 1D) in mice following IRI corresponding to the kidney dysfunction and proximal tubular necrosis (Fig. S1). *In situ* hybridization (ISH) with *Fg*α, *Fgβ* and *Fgγ* in the liver tissue revealed strong diffuse cytoplasmic staining in the hepatocytes (Fig. 1B, biological positive control). In the kidney, the staining varied in intensity and distribution between the chains and with respect to the presence or absence of injury (Fig. 1B, first two columns). *Fgα* in uninjured (sham) kidney revealed diffuse cytoplasmic staining that was less intense than the reactivity in liver under the same conditions. The staining was more intense and perinuclear in distribution 24 h after IRI. The staining was more intense and perinuclear in distribution 24 h after IRI. ISH for *Fgβ* in uninjured kidney was as intense as in liver, but revealed more intense diffuse cytoplasmic distribution at 24 h. ISH for *Fgγ* was of similar reactivity to *Fgα* in distribution and intensity, in uninjured and injured kidney; there was more diffuse cytoplasmic staining in uninjured tissue, with less intense reactivity when compared to the liver tissue, but in IRI, the reactivity intensified and distributed around the nuclei, similar to the *Fgα*.

Phenotypically the *Fg^+/+^*, and mutant mice for *FgA-α* chain [heterozygous (*Fg^+/−^*) or knockout (*Fg^−/−^*)] demonstrated following features (Fig. S2A): i) loss of *Fgα* chain with, albeit modestly decreased, but detectable transcription and translation of *Fgβ* and *Fgγ* chains in the liver of *Fg^−/−^* mice (Fig. S2B) as reported previously [9]; ii) significant decrease in transcription and translation of *Fgα* and *Fgβ* with no alterations in *Fgγ* chain in the kidney of *Fg^−/−^* mice (Fig. S2C) as compared to *Fg^+/+^* mice; iii) approximately 50% decrease in plasma Fg D-Dimer levels in *Fg^+/−^* mice and undetectable Fg protein in the circulation in *Fg^−/−^* mice as compared to *Fg^+/+^* (Fig. S2D); iv) unaltered urinary Fg protein excretion (Fig. S2E).

**Figure 2:**
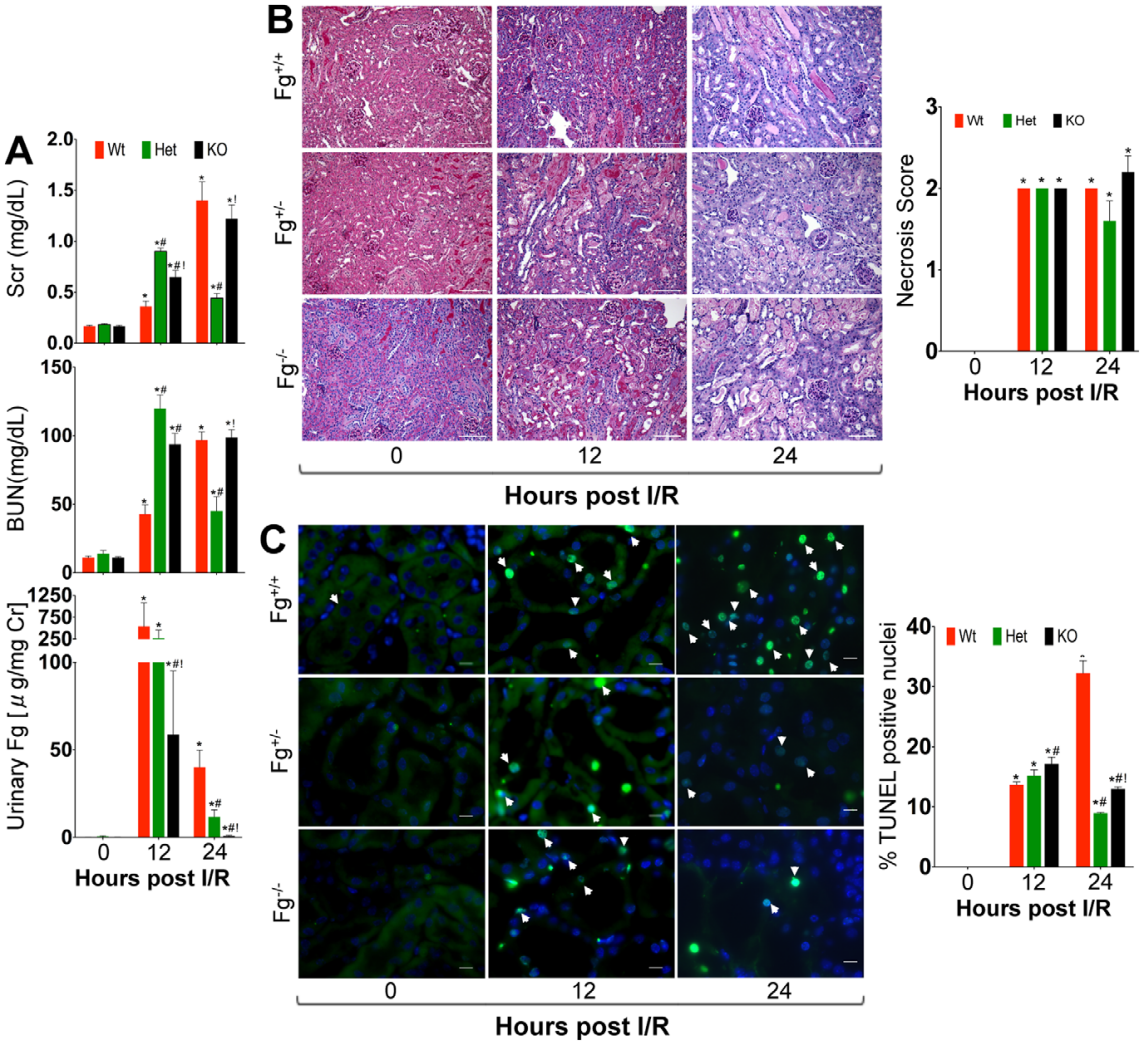
Fibrinogen heterozygosity protects from progression of kidney dysfunction and apoptosis following IRI. Male wild type (*Fg^+/+^*), heterozygous *(Fg^+/−^*) and knock out (*Fg^−/−^*) mice were subjected to IRI and assessed for kidney injury parameters (n = 6/group/time point) A) Serum creatinine (Scr), Blood Urea Nitrogen (BUN) and urinary Fg levels. B) Representative histopathological images using periodic acid-schiff (PAS) staining and quantitative necrosis score represented graphically on the right. C) Apoptotic cells (green) determined by TUNEL assay. Percentage of positive staining TUNEL nuclei is represented graphically on the right of photomicrographs. *represents p<0.05 in comparison to sham; ^#^represents p<0.05 as compared to wild type within the time point and ^!^represents p<0.05 as compared to heterozygous within the time point as determined by one-way ANOVA. Bar represents 10 µm.

Following IRI, *Fg^+/−^* and *Fg^−/−^* mice showed greater kidney dysfunction as assessed by BUN and SCr (Fig. 2A) at 12 h as compared to *Fg^+/+^* mice (Fig. 2A). However, by 24 h the kidney dysfunction in the *Fg^+/+^* and *Fg^−/−^* mice further escalated whereas, the *Fg^+/−^* mice appeared to show rapid functional restoration with BUN and SCr levels ∼2–3 fold lower than the *Fg^+/+^* and *Fg^−/−^* mice (Fig. 2A). Urinary Fg, serving as a sensitive indicator of kidney tubular injury [21], also showed a massive increase at 12 h in all three groups but significantly reduced levels in *Fg^+/−^* (∼5-fold) and Fg^−/−^ (∼10- fold) mice at 24 h as compared to *Fg^+/+^* mice (Fig. 2A). Kidney histology showed severe necrosis in the cortico-medullary junction of all post ischemic kidneys particularly in the S3 segments at 12 and 24 h (Fig. 2B). Although differences between the *Fg^+/+^*, *Fg^+/−^* and *Fg^−/−^* mice in the extent of necrosis were subtle, there was a marked reduction in the number of apoptotic cells at 24 h in the *Fg^+/−^* and *Fg^−/−^* mice as compared to *Fg^+/+^* mice (Fig. 2C).

**Figure 3:**
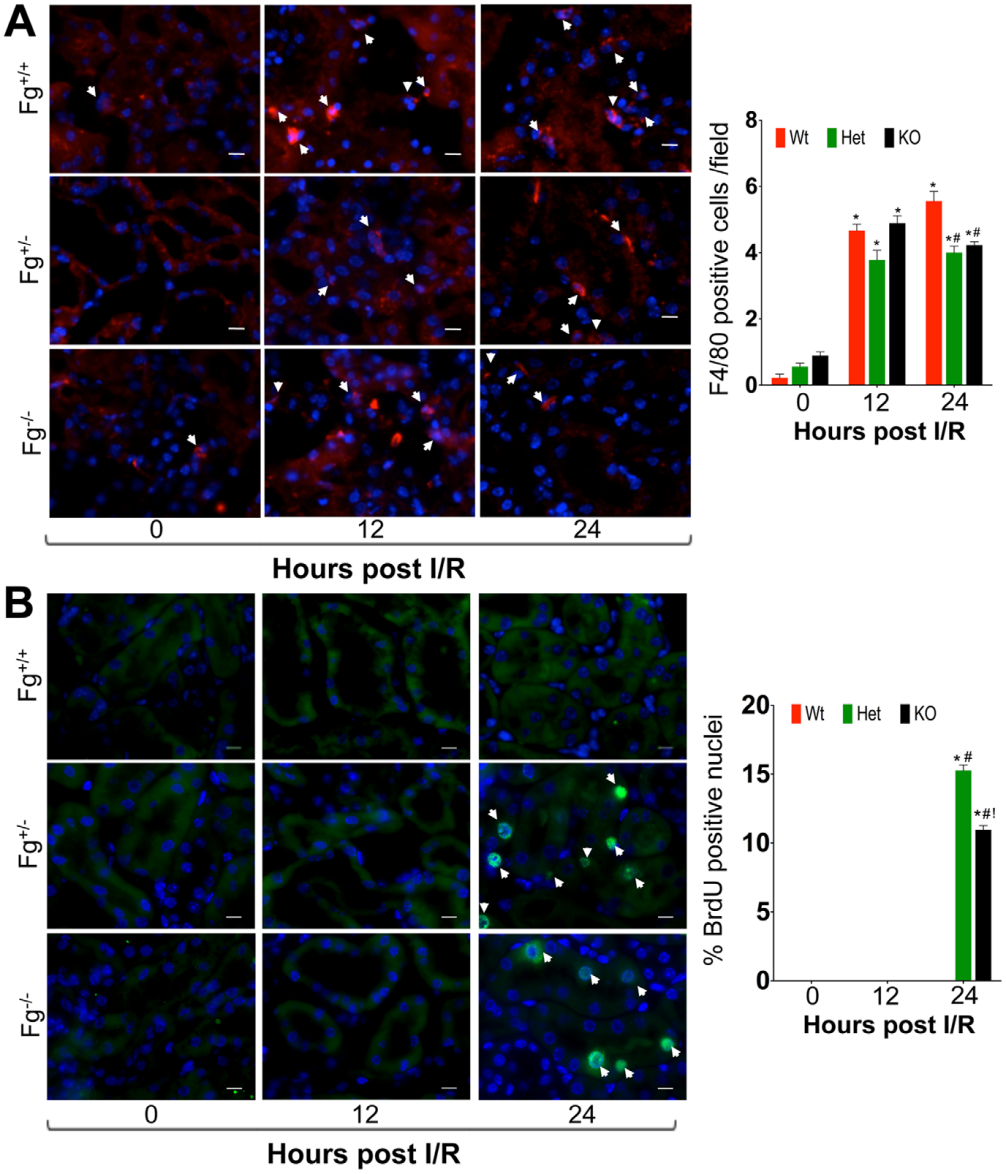
Heterozygous and knockout Fg mice exhibit efficient immune cell clearance coupled with robust tubular epithelial cell proliferation. Fixed frozen sections following IRI were stained for A) macrophage F4/80 (red). Number of F4/80 cells per 60X field is represented graphically on the right of the photomicrograph. B) BrdU positive cells (green) by immunofluorescence. Percentage of positive staining for BrdU positive nuclei is represented graphically on the right of photomicrographs. Arrowheads indicate positive cells/nucleus and bar represents 10 µm. *represents p<0.05 in comparison to sham; ^#^represents p<0.05 as compared to wild type within the time point and ^!^represents p<0.05 as compared to heterozygous within the time point as determined by one-way ANOVA. Bar represents 10 µm.

We next evaluated the contribution of inflammatory immune cell invasion in progression of kidney injury by immunostaining for markers of macrophage (F4/80) and neutrophil (Ly-6G) infiltration. The number of F4/80 positive cells was similar at 12 h in all three groups but at 24 h the *Fg^+/−^* and *Fg^−/−^* mice showed a statistically significant decrease in the number of macrophages (Fig. 3A) as well as Ly-6G positive neutrophils (Fig. S3) as compared to *Fg^+/+^* mice.

To evaluate the kidney tissue repair we quantitated the number of proliferating epithelial cells by BrdU immunostaining and found that *Fg^+/−^* and *Fg^−/−^* mice exhibited significantly greater number of BrdU positive cells as compared to *Fg^+/+^* mice at 24 h (Fig. 3B). This suggests that although the initiation and early phase of injury was similar in all three groups there was a timely and efficient tissue repair response in the *Fg^+/−^* and *Fg^−/−^* mice, which curbed inflammation and apoptosis resulting in regression of injury. We hypothesized that in the *Fg^+/+^* mice there is progression of the initial injury because of Fg binding to Intercellular Adhesion Molecule-1 (ICAM-1) that promotes apoptosis and cell death through ERK phosphorylation.

**Figure 4:**
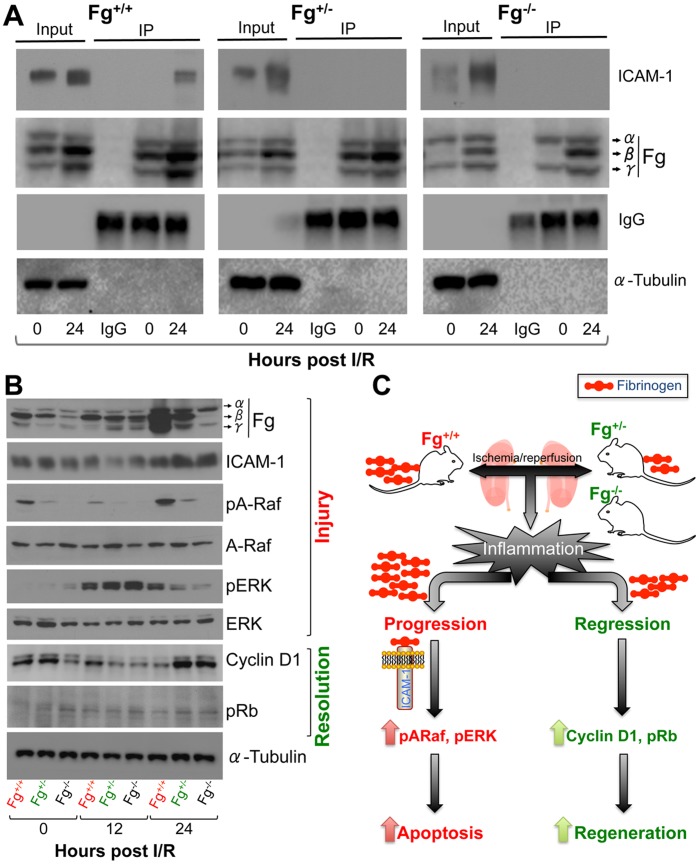
Fibrinogen binds to ICAM-1 in the kidney leading to sustained tissue injury. A) Kidney tissue lysates were immunoprecipitated with anti-fibrinogen antibody and immunoblot was performed for ICAM-1. IgG light chain served as loading control for IP and β-Actin served as loading control for input. B) Following 29 min IRI kidney tissue lysates were prepared and equal protein was resolved on SDS-PAGE for western blot analysis for pERK, ERK, Cyclin D1 and pRb. α-Tubulin served as loading control. C) Schematic showing that in the Fg^+/−^ mice a reduction in availability of excess Fg from interacting with ICAM-1 prevents progression of injury thereby allowing timely induction of Cyclin D1 and pRb mediating efficient kidney tissue repair and resolution of injury.

We found that there was significant binding of Fg to ICAM-1 in kidney tissues of *Fg^+/+^* mice at 24 h following IRI as compared to a complete absence of binding observed in the *Fg^+/−^* and *Fg^−/−^* mice (Fig. 4A). Consistently there was a significant decrease in expression of Fg protein at 24 h in *Fg^+/−^* and *Fg^−/−^* mice kidneys as compared to *Fg^+/+^* mice following IRI (Fig. 4B). We further confirmed Fg binding to ICAM-1 in tubular epithelial cells by co-immunostaining that showed a Pearson co-localization coefficient between Fg and ICAM-1 to be 0.6 in *Fg^+/+^* as compared to 0.4 and 0.05 in *Fg^+/−^* and *Fg^−/−^* respectively (Fig. S4). Raf-1 and ERK were highly activated as evident by significantly higher phosphorylation in the *Fg^+/+^* kidneys at 24 h following IRI as compared to *Fg^+/−^* and *Fg^−/−^* mice kidneys. pERK was upregulated at 12 h in all three groups confirming the earlier observations (Fig. 2) about similar level of necrosis, apoptosis and inflammation at 12 h in the three groups of mice. On the other hand Cyclin D1 and pRb, indicating higher cell proliferation, were significantly greater at 24 h in the *Fg^+/−^* and *Fg^−/−^* as compared to *Fg^+/+^* kidneys (Fig. 4B).

## Discussion

Our results support the hypothesis that a reduction in availability of excess Fg from interacting with ICAM-1 prevents progression of injury thereby allowing timely induction of Cyclin D1 and pRb mediating efficient kidney tissue repair and resolution of injury (Fig. 4C). This hypothesis is consistent with the previous reports demonstrating therapeutic potential of Fg-derived peptides (Bβ_15–42_ and γ_377–395_) by competing with native Fg for binding to vascular endothelial cadherin (VE-cadherin), ICAM-1, CD11b/CD18 which in turn inhibits infiltration of leukocytes at the site of injury and prevents exacerbation of injury [18], [23]. Furthermore, others and we have previously shown that Bβ_15–42_ peptide administration protected from kidney IRI by increasing tissue repair and decreasing apoptosis [21], [24] confirming that pharmacological reduction in excess Fg paves the way for faster structural and functional recovery.

Thus, the new findings of this study are i) Fg (*Fgα*, *Fgβ*, and *Fgγ*) is transcribed in the kidney and its mRNA levels, protein expression and urinary excretion significantly increase following IRI; ii) Heterozygosity of mouse FgAα chain results in global reduction of Fg production to a moderate level that protects the *Fg^+/−^* mice from IRI-induced kidney tubular injury, kidney dysfunction, inflammation and apoptosis by launching an efficient tissue regeneration response. Although *Fg^−/−^* mice showed a reduction in apoptosis, reduced immune cell infiltration and increased regeneration, the functional and structural restoration of the kidney after IRI was not as rapid as *Fg^+/−^* mice potentially due to the impedance with clotting.

Fibrinogen binding to ICAM-1 through its γ_117–133_ domain has been well documented on endothelial cells [25], [26] and has been shown to promote leukocyte transmigration by acting as an intermediary molecule that can bind both ICAM-1 and leukocytes through the Mac-1 receptor [27]. We extended these studies and show that Fg binds to ICAM-1 in the kidney following IRI thereby potentially activating Raf-1, triggering the Raf-MEK-ERK pathway, which in turn can activate an apoptotic response. Experiments using anti-ICAM-1 antibodies as well as ICAM-1–deficient mice have shown ICAM-1 to be a key mediator of acute IRI injury via potentiation of neutrophil–endothelial interactions [28]. ICAM-1 expression significantly increases on proximal tubular epithelial cells in patients with acute renal allograft rejection [29] and here we found significant co-localization of ICAM-1 with Fg (Pearson’s co-localization coefficient of 0.6, Fig. S4) predominantly on the proximal tubular epithelial cells emphasizing the paradigm that tubular epithelium is not merely a passive victim of injury but also an active participant in the inflammatory response in kidney IRI [30].

In summary, our experiment shows that kidney expresses Fgα, Fgβ and Fgγ transcripts and genetic manipulation resulting in decreased availability of Fg protein to interact with cellular receptors diminishes the molecular response cascade and dampens the inflammatory response leading to faster resolution of injury.

## Supporting Information

Figure S1 **Characterization of kidney dysfunction and tubular injury following bilateral renal ischemia/reperfusion injury (IRI).** Male *BALB/c* were subjected to IRI and kidneys, blood and urines were collected over time (n = 5/time point). A) Serum creatinine (Scr) and Blood Urea Nitrogen (BUN) measurements. B) Representative histological H&E stained images following IRI at 24, 48 and 72 h showing proximal tubular necrosis as compared to sham. Bar represent 100 µm.
(TIF)

Figure S2 **Genotype and phenotype characterization of Fg wild type, heterozygous and knockout mice.** A) Genotyping results from a representative group (n = 7) of Fg wild type, heterozygous and knockout mice as described in methods section. B) Real time PCR and Western Blot analysis for Fg (*Fgα*, *Fgβ* and *Fgγ*) in the liver and C) Kidney of Fg wild type, heterozygous and knockout mice. D) Plasma levels of Fg in wild type, heterozygous and knockout mice were measured by D-Dimer ELISA test and by western blot analysis. E) Urinary levels of Fg were measured using a Luminex based assay in wild type, heterozygous and knockout mice. *represents p<0.05 as determined by student’s t-test in comparison to wild type.
(TIF)

Figure S3 **Heterozygous and knockout Fg mice show significantly decreased neutrophil infiltration following IRI.** Fixed frozen section following IRI at 12 and 24 h were stained for Ly-6G (green). Number of Ly-6G positive nuclei is represented graphically on the right of photomicrographs. Arrowheads indicate neutrophils and bar represent 10 µm. *represents p<0.05 in comparison to sham; ^#^represents p<0.05 as compared to wild type within the time point and ^!^represents p<0.05 as compared to heterozygous within the time point as determined by one-way ANOVA. Bar represent 10 µm.
(TIF)

Figure S4 **Significant colocalization of Fg and ICAM in the kidney following IRI.** Fixed frozen section following IRI at 12 and 24 h were co-stained for Fg (red) and ICAM-1 (green). Pearson’s coefficient was plotted as a measure of co-localization on the right of photomicrographs. *represents p<0.05 in comparison to sham; ^#^represents p<0.05 as compared to wild type within the time point and ^!^represents p<0.05 as compared to heterozygous within the time point as determined by one-way ANOVA. Bar represent 10 µm.
(TIF)

Table S1 **Primer sequences for genotyping and Real Time PCR analysis for candidate genes.**
(DOC)
