# Supplementary figures and images for: Heterozygosity for Fibrinogen Results in Efficient Resolution of Kidney Ischemia Reperfusion Injury
Source: PLoS One. 2012 Sep 19;7(9):e45628. doi: 10.1371/journal.pone.0045628 (PMC3446934; doi:10.1371/journal.pone.0045628)

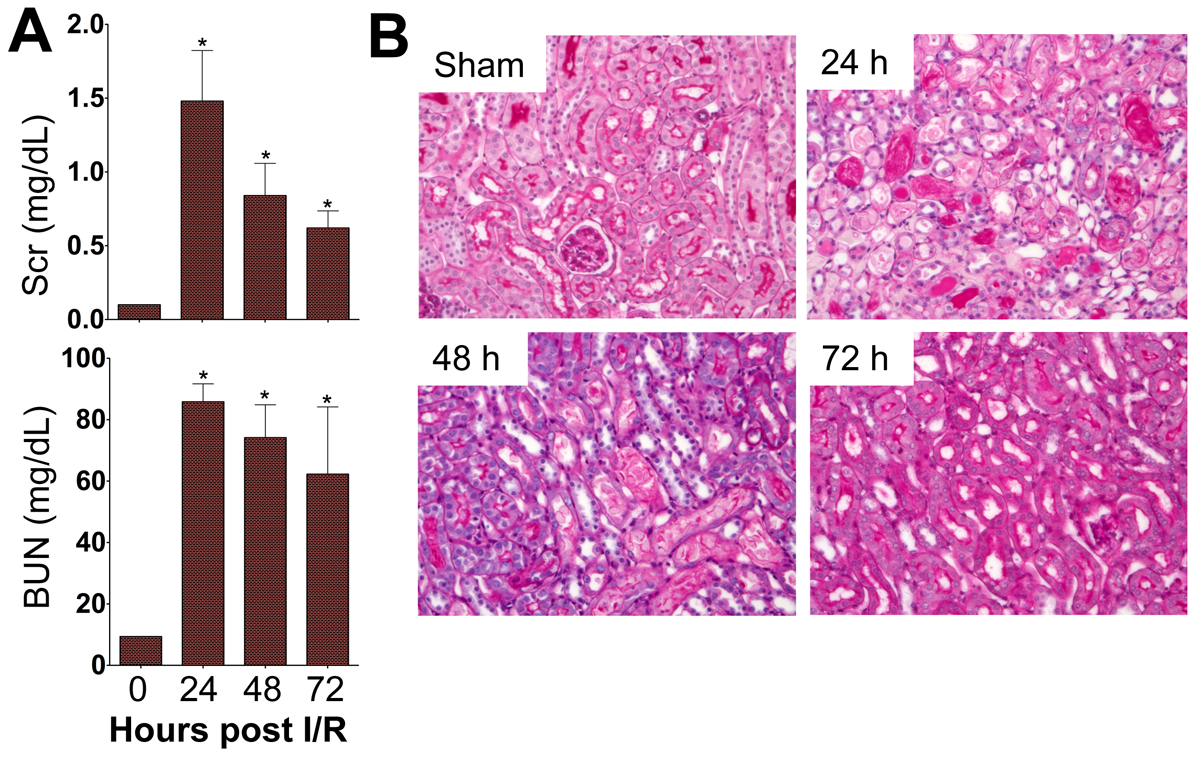

Supplement: Figure S1 — Characterization of kidney dysfunction and tubular injury following bilateral renal ischemia/reperfusion injury (IRI). Male BALB/c were subjected to IRI and kidneys, blood and urines were collected over time (n = 5/time point). A) Serum creatinine (Scr) and Blood Urea Nitrogen (BUN) measurements. B) Representative histological H&E stained images following IRI at 24, 48 and 72 h showing proximal tubular necrosis as compared to sham. Bar represent 100 µm. (TIF) [file pone.0045628.s001.tif]

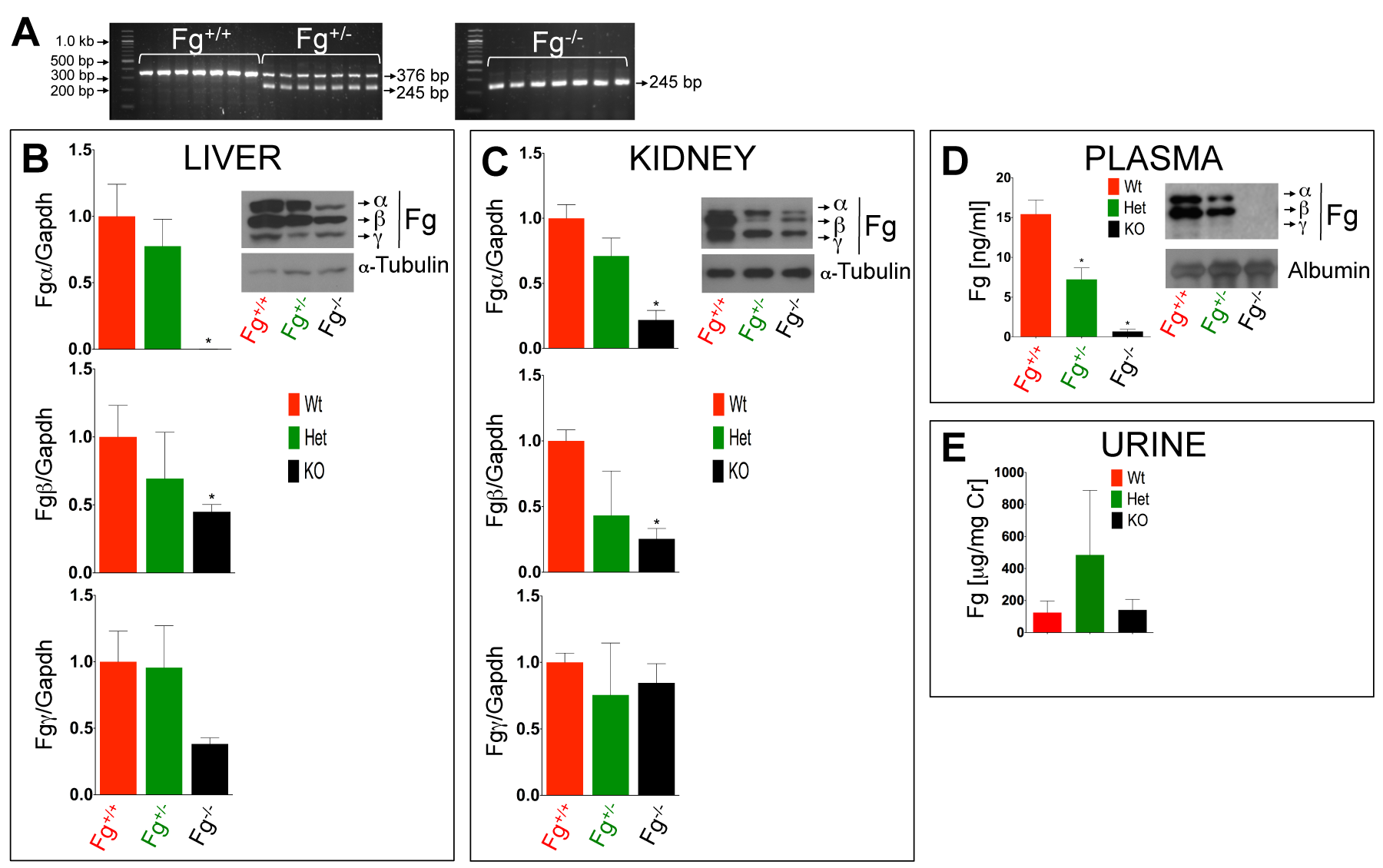

Supplement: Figure S2 — Genotype and phenotype characterization of Fg wild type, heterozygous and knockout mice. A) Genotyping results from a representative group (n = 7) of Fg wild type, heterozygous and knockout mice as described in methods section. B) Real time PCR and Western Blot analysis for Fg (Fgα, Fgβ and Fgγ) in the liver and C) Kidney of Fg wild type, heterozygous and knockout mice. D) Plasma levels of Fg in wild type, heterozygous and knockout mice were measured by D-Dimer ELISA test and by western blot analysis. E) Urinary levels of Fg were measured using a Luminex based assay in wild type, heterozygous and knockout mice. *represents p<0.05 as determined by student’s t-test in comparison to wild type. (TIF) [file pone.0045628.s002.tif]

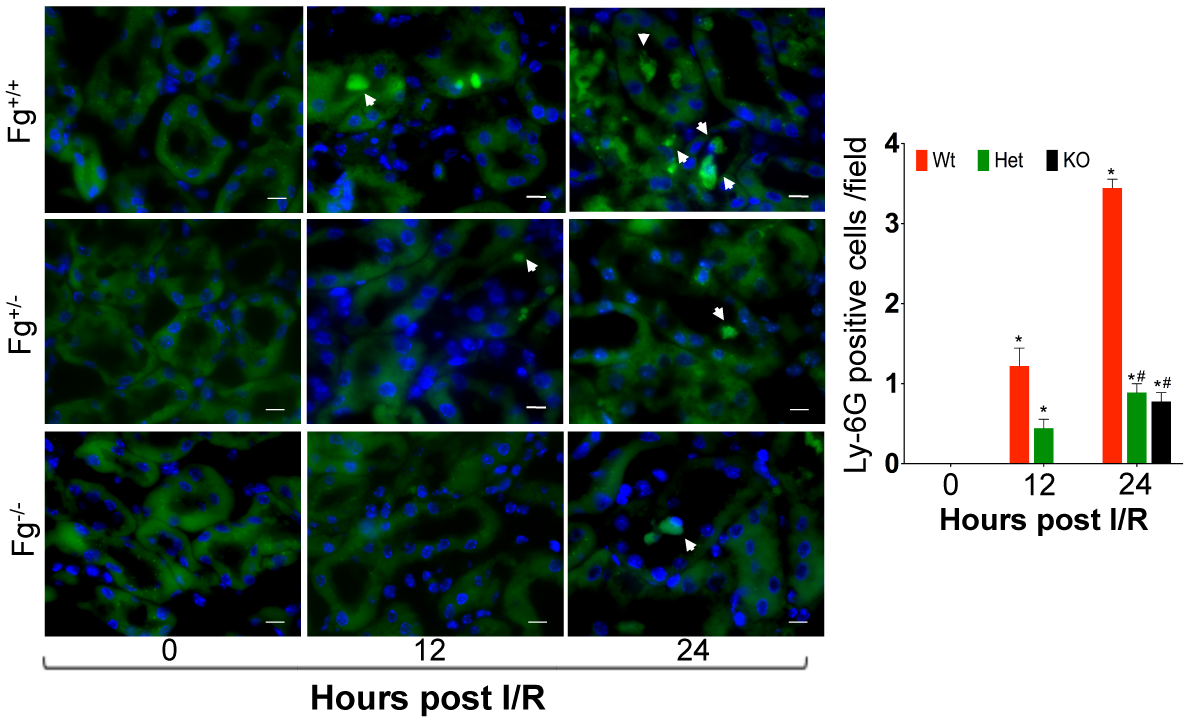

Supplement: Figure S3 — Heterozygous and knockout Fg mice show significantly decreased neutrophil infiltration following IRI. Fixed frozen section following IRI at 12 and 24 h were stained for Ly-6G (green). Number of Ly-6G positive nuclei is represented graphically on the right of photomicrographs. Arrowheads indicate neutrophils and bar represent 10 µm. *represents p<0.05 in comparison to sham; #represents p<0.05 as compared to wild type within the time point and !represents p<0.05 as compared to heterozygous within the time point as determined by one-way ANOVA. Bar represent 10 µm. (TIF) [file pone.0045628.s003.tif]

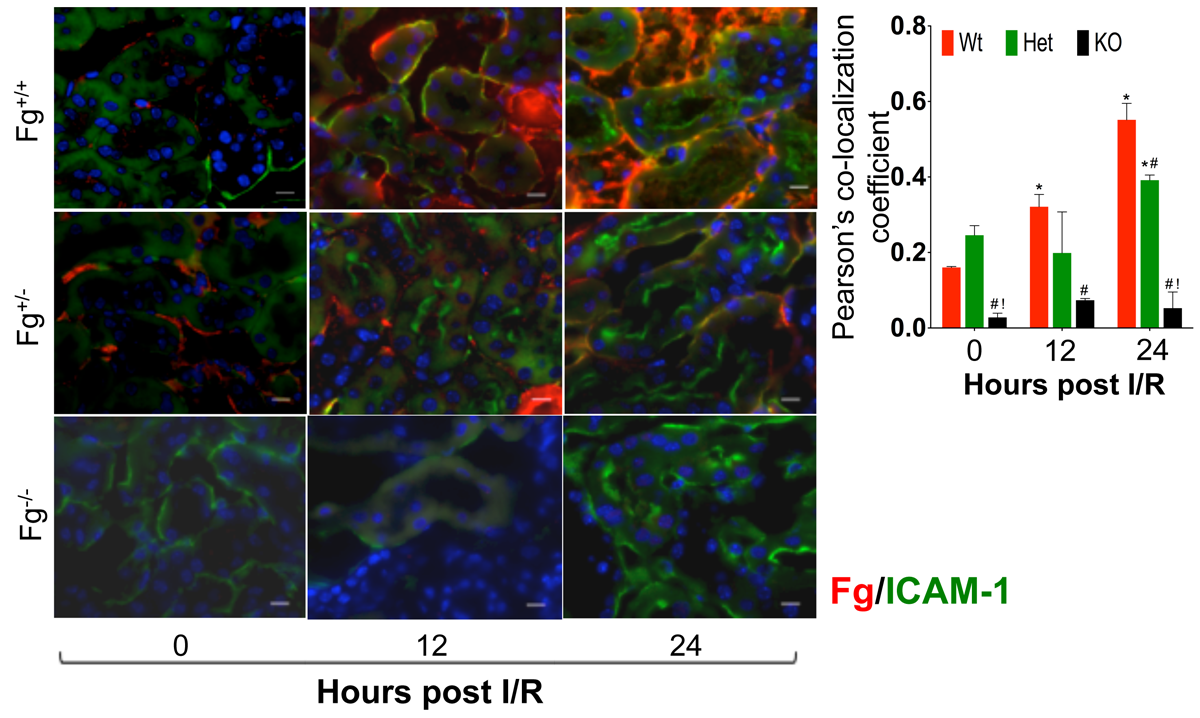

Supplement: Figure S4 — Significant colocalization of Fg and ICAM in the kidney following IRI. Fixed frozen section following IRI at 12 and 24 h were co-stained for Fg (red) and ICAM-1 (green). Pearson’s coefficient was plotted as a measure of co-localization on the right of photomicrographs. *represents p<0.05 in comparison to sham; #represents p<0.05 as compared to wild type within the time point and !represents p<0.05 as compared to heterozygous within the time point as determined by one-way ANOVA. Bar represent 10 µm. (TIF) [file pone.0045628.s004.tif]
