# Supplementary material for: Heterozygosity for Fibrinogen Results in Efficient Resolution of Kidney Ischemia Reperfusion Injury
Source: PLoS One. 2012 Sep 19;7(9):e45628. doi: 10.1371/journal.pone.0045628 (PMC3446934; doi:10.1371/journal.pone.0045628)
Supplement: Table S1 — Primer sequences for genotyping and Real Time PCR analysis for candidate genes. (DOC) [file pone.0045628.s005.doc]

**Table S1. Primer sequences for genotyping and Real Time PCR analysis for candidate genes.**

| **Gene** | **F/R** | **Sequence** |
| --- | --- | --- |
| *Genotyping* | | |
| *FIBA* | F | 5’- TGC TGG ATC AAT CCC CAG CAA CCG TGA GAG |
|  | R | 5’- GCT TCA GCT CCA GTT CTC CTC ATG AGC CAT |
| *HPRT* | F | 5’- TAT TAC CAG TGA ATC TTT GTC AGC AG |
| *Real-Time PCR of Candidate Genes* | | |
| *Fgα* | F | 5’-TGT GGA GAG ACA TCA GAG TCA ATG |
|  | R | 5’-CGT CAA TCA ACC CTT TCA TCC |
| *Fgβ* | F | 5’-CTA TGG CTG CTG CTG CTA TTG |
|  | R | 5’-GGC TCT TCC TTT CTC CTG TCA AC |
| *Fgγ* | F | 5’-TGT GGC TAC CAG AGA TAA CTG TTG |
|  | R | 5’-ATG TCT TCC AGC GTT CGG AG |
| *GAPDH* | F | 5’-GAA TAC GGC TAC AGC AAC AGG |
|  | R | 5’-GGT CTG GGA TGG AAA TTG TG |
